# Does phenotypic plasticity initiate developmental bias?

**DOI:** 10.1111/ede.12304

**Published:** 2019-07-26

**Authors:** Kevin J. Parsons, Kirsty McWhinnie, Natalie Pilakouta, Lynsey Walker

**Affiliations:** ^1^ Institute of Biodiversity, Animal Health, and Comparative Medicine University of Glasgow Glasgow UK

**Keywords:** cryptic genetic variation, developmental signaling pathways, flexible stem, plasticity integration

## Abstract

The generation of variation is paramount for the action of natural selection. Although biologists are now moving beyond the idea that random mutation provides the sole source of variation for adaptive evolution, we still assume that variation occurs randomly. In this review, we discuss an alternative view for how phenotypic plasticity, which has become well accepted as a source of phenotypic variation within evolutionary biology, can generate nonrandom variation. Although phenotypic plasticity is often defined as a property of a genotype, we argue that it needs to be considered more explicitly as a property of developmental systems involving more than the genotype. We provide examples of where plasticity could be initiating developmental bias, either through direct active responses to similar stimuli across populations or as the result of programmed variation within developmental systems. Such biased variation can echo past adaptations that reflect the evolutionary history of a lineage but can also serve to initiate evolution when environments change. Such adaptive programs can remain latent for millions of years and allow development to harbor an array of complex adaptations that can initiate new bouts of evolution. Specifically, we address how ideas such as the flexible stem hypothesis and cryptic genetic variation overlap, how modularity among traits can direct the outcomes of plasticity, and how the structure of developmental signaling pathways is limited to a few outcomes. We highlight key questions throughout and conclude by providing suggestions for future research that can address how plasticity initiates and harbors developmental bias.

## 1 DOES PHENOTYPIC PLASTICITY INITIATE DEVELOPMENTAL BIAS?

Darwin (1859, p. 31, 80–82, 127–131, 170) posited that phenotypic variation is generated randomly, and selection acts to sort such variation to cause adaptation. While Darwin was unaware of the mechanisms of inheritance, the theory of natural selection is now based on the idea that random genetic mutation provides the source of new selectable variation (i.e., neo‐Darwinian thinking). This focus has led to other sources of variation being previously relegated as “problem variables” incapable of contributing to adaptive evolution (see Falconer, 1952). However, *selection*, as viewed by Darwin, does not specifically require genetic variation, rather it only needs variable fitness among phenotypes to operate and sort variation (Mayr, 1997). Darwin's version of *evolution* via selection requires only heritability of these sorted phenotypes to persist over generations. Thus, in Darwin's view, the source of variation for selection can be variable, and while he was naïve to genetic variation, it means variation that is generated outside a simple “genetic mutation‐to‐phenotypic‐variant” process can contribute toward adaptative evolution. Indeed, among nongenetic sources of variation, a plethora of data now support the idea that environmentally induced phenotypes can provide variation for selection via phenotypic plasticity (Levis & Pfennig, 2016; Parsons, Sheets, Skúlason, & Ferguson, 2011; Parsons et al., 2016; Sultan, 2015). While sources of variation for adaptive evolution are being broadened (see Bailey, Rodrigue, & Kassen, 2015; Sultan, 2015; Bonduriansky & Day, 2018), nature by which this variation is produced has rarely been addressed. Specifically, Darwin's idea that variation is generated randomly has largely been taken for granted rather than tested, representing a fundamental gap in our understanding of evolution.

Although once relegated as one of the “problem variables,” plasticity as a provider of variation has become well accepted within evolutionary biology over the past 20 years. In many ways, plasticity is now seen as a conventional trait possessing heritable variation that is widely evident among the reaction norms of different clonal lines and across populations (Pigliucci, 2006; Skúlason et al., 2019). This allows selection to favor relative increases in fitness and act upon *environmentally induced* phenotypes to alter the frequency of alleles that modify reaction norms (Gomez‐Mestre & Jovani, 2013). In this sense, plastic responses are often seen as a way of development to uncover genetic variation in the phenotype and therefore expose it to selection. Thus, the evolution of adaptive plasticity is seen as being well in line with a neo‐Darwinian process with the generation of random mutations accompanying random effects on reaction norms and providing fodder for natural selection. In fact, there is a growing view that plasticity is often responsible for the initiation of adaptive divergence (i.e., “plasticity‐first” evolution), leading the way for genetic variation to follow (Levis & Pfennig, 2016; West‐Eberhard, 2003).

Plasticity has had a relatively rapid shift into standard evolutionary thinking relative to other concepts proposed for the extended evolutionary synthesis (see Pigliucci, 2007; Ledón‐Rettig, Pfennig, Chunco, & Dworkin, 2014). The core to this acceptance probably lies in the idea that plasticity can be viewed as a property of the genotype (see Pigliucci, 2006), which allows it to fit the conventions of straightforward *genetic* heritability that forms the core of neo‐Darwinism. Indeed, plasticity itself is usually defined as an ability of a genotype to respond to environmental cues to produce a phenotypic variation. This definition of plasticity should be considered “gene‐centric” serving as a way to translate (but also overlook) the complexities of development to fit plasticity to a neo‐Darwinian paradigm. Instead, it may be that plasticity is more accurately defined as the property of development and that we need to embrace the inherent complexities of this view (e.g., threshold responses, nonlinear interactions) to gain a more complete understanding of how it contributes variation to evolution. This is a burgeoning issue, as we already know that development can be affected by more than simply the genotype and the environmental conditions of the current generation. For example, parental or transgenerational effects can be transduced by parents in utero or in vivo to alter a wide array of phenotypes including the reaction norms of plastic responses (Nettle & Barton, 2015; Sultan, 2015). Further, genetic mutations occur within the context of developmental systems that do not allow them to operate in isolation to directly affect the phenotype. Instead, our emerging understanding from evo–devo and systems biology makes it clear that adaptive variation is often generated by interactions within molecular signaling pathways that are comprised of several genes (Moczek et al., 2015; Murray, 2018). Such interactions within developmental systems can be functionally redundant and play an important role in funneling variation toward similar outcomes and, in turn, biasing the patterns and levels of phenotypic variation that are exposed to natural selection. Thus, the random generation of phenotypic variation posited by Darwin (1859) may not apply to cases of plasticity or even standard mutations.

Here, we focus on developmental biases that may arise through phenotypic plasticity. We define developmental bias as the tendency of developmental systems to generate a nonrandom variation. Taking the view that plasticity is a property of developmental systems, we first explain how in some contexts, plasticity may drive biased developmental responses in the phenotype. We then move on to the inverse situation whereby developmental responses may bias plasticity. Last, we discuss how such processes interact to contribute to the evolution and lead to biased but adaptive phenotypic outcomes. While we draw on a number of examples across study systems, we also focus on patterns of similarity in the craniofacial variation of fishes as a case study for the role of plasticity in generating bias in microevolution and macroevolution. Fishes have been the object of intense investigation at a number of biological levels, allowing for connections among processes to be inferred.

## 2 PLASTICITY‐DRIVING DEVELOPMENTAL BIAS THROUGH ACTIVE RESPONSES AND THE FLEXIBLE STEM

It is important to distinguish between cases where organisms are adapted to use specific environmental cues to dictate development, and cases where an organism is simply passively responding to a variable environment. To address this, over 36 years ago, Smith‐Gill (1983) proposed two different conceptual forms of phenotypic plasticity: active and passive. Active plasticity is considered anticipatory, likely based on the previous history in a lineage, and can enhance phenotype–environment matching to increase fitness. For example, bone growth and remodeling is locally responsive to and accentuated by mechanical stress (Hall, 2015). Therefore, functional demands on the skeleton that vary with the environment should, in turn, result in changes in form that are often localized and nonrandom in their distribution (and relate to function). In contrast, passive plasticity may involve ubiquitous changes in metabolism and growth that involve a range of mechanisms and may result in somewhat random phenotypic responses to environmental inputs, often assumed to be due to resource limitations. Passive responses, unlike active ones, are not necessarily adaptive and can involve maladaptive responses at any stage of life history, whereas the phenotypic variation produced by passive responses is nonspecific (Smith‐Gill, 1983). Together, these aspects of passive plasticity offer no specific driver for change in a developmental program. Though active and passive forms of plasticity have often been suggested as being difficult to distinguish empirically (West‐Eberhard, 2003), it is probably the case that any plastic response is an amalgamation of such conceptual forms of reaction. However, in many cases where selection has been operating, it is probable that responses are dominated by active responses which may bias the phenotypic outcomes of plasticity. This could be indicated by similar responses to similar environments across species and populations.

Across populations and species, such biased phenotypic responses would be accompanied by similar functional outcomes that can be adaptive. This has been particularly well‐characterized in response to certain environmental gradients such as shade avoidance responses in plants, whereby a “shade avoidance syndrome” occurs and is thought to be adaptive for growth and development in high plant densities (Martinez‐Garcia et al., 2014). These responses can be elicited by variation in proximity and in response to direct plant canopy shade. As vegetation preferentially reflects far‐red (FR) light compared to other wavelengths, plant proximity generates a reduction in the red (R, about 600–700 nm) to far‐red (FR, between 700–800 nm) ratio (R:FR) in the light impinging on neighbors. This low R:FR signal is perceived by the phytochrome photoreceptors and has a major role in controlling several adaptive responses such as seed germination, stem elongation, leaf expansion, and flowering time. Though these responses have been demonstrated to be adaptive (Callahan & Pigliucci, 2002; Schmitt, McCormac, & Smith, 1995), it is an open question whether these responses contribute to evolutionary trends in life history and morphological characteristics among plant populations and species.

**Figure 1:**
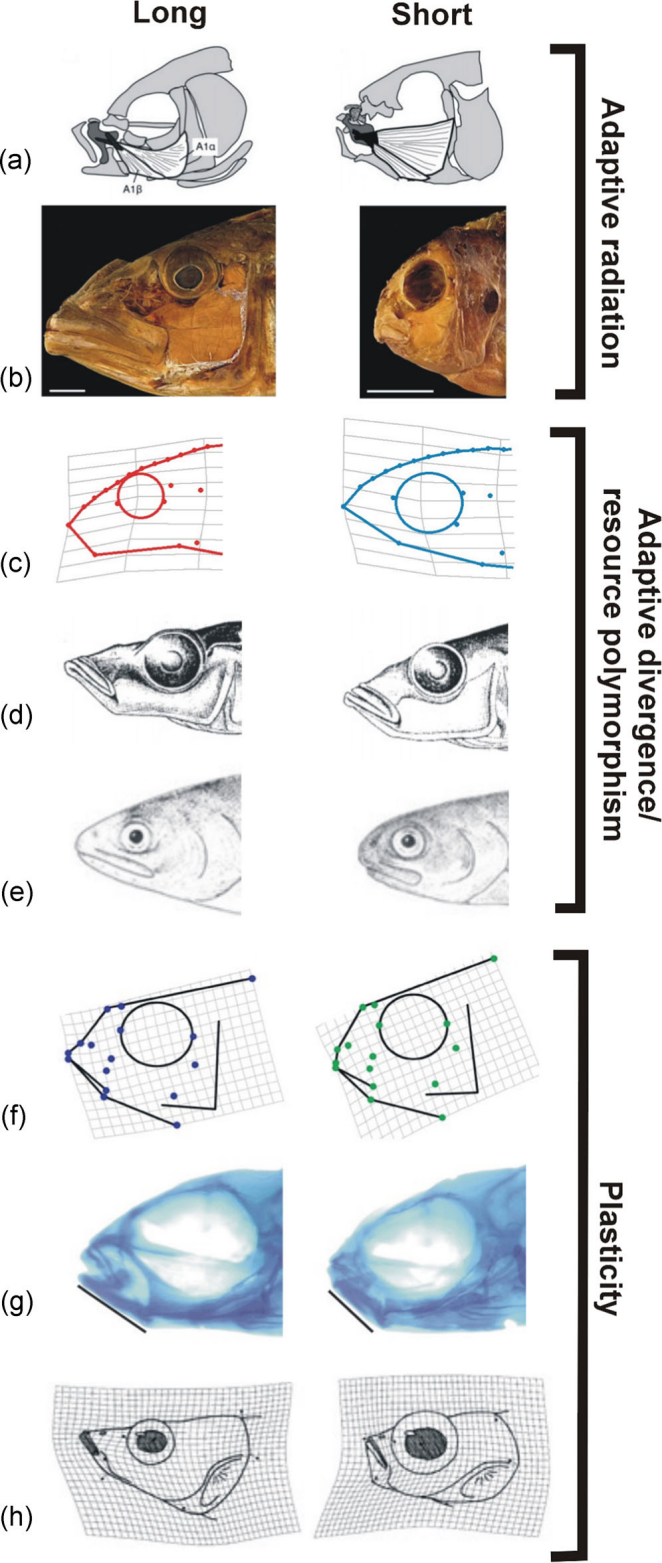
The preorbital region of fishes appears to possess developmental bias affecting its size and length. This is apparent across examples of adaptive radiation, population‐level adaptive divergence or resource polymorphism, and in morphological plasticity. In some cases, patterns of evolution match patterns of plasticity. Also, plasticity can show a similar pattern of response in spite of widely different environmental cues. In (a,b), we see that major trends in the adaptive radiations of cyprinids comprising over 3,000 (adapted from Hernandez & Staab, 2015) and African cichlids comprising over 1,200 species (adapted from Cooper et al., 2010) respectively, involve changes in the preorbital region of the craniofacial apparatus. Similarly, at the level of populations in (c,e), we see that adaptive divergence or resource polymorphisms involve changes in the preorbital region. Specifically, in (c,d), we see two cases of divergence in three‐spined sticklebacks showing a similar change in preorbital length. However, in (c), divergence is occurring along a thermal habitat gradient between geothermally warmed and ambient populations (red = warmed, blue = ambient; Pilakouta et al., 2019), whereas in (d) divergence is occurring along a limnetic (long side) and benthic (short side) gradient (image credit, Elizabeth Carefoot). In (e), planktivorous (long side) and benthivorous (ecomorphs) of arctic charr (*Salvelinus alpinus*) from Lake Thingvallavatn, Iceland are depicted (drawn by Eggert Petursson). In (f,g), we see the outcome of plasticity experiments, with the effects of limnetic (long side) and benthic (short side) foraging treatments in F3 hybrid cichlids (from Parsons et al., 2016). In (g), we see the morphological responses of juvenile sticklebacks to rearing at 18°C (long side) and 12°C (short side) (Campbell and Parsons, unpublished), which follow a similar pattern to limnetic (long side) and benthic (short side) treatments in sticklebacks depicted in (g); (from Wund et al., 2008) [Color figure can be viewed at wileyonlinelibrary.com]

In fishes, adaptive divergence often occurs between benthic and limnetic habitats. This includes several adaptive radiations and divergence in fishes whereby species differ in craniofacial morphology on the basis of habitat use (Cooper & Westneat, 2009; Cooper et al., 2010). Specifically, fish that feed on benthic prey tend to have relatively shorter jaws, which lead to an increased mechanical advantage. This is particularly useful for foraging on surfaces and applying a strong bite force such as for algae grazing or crushing snails. Fish that feed on limnetic prey tend to have longer jaws which increase the volume of the oral cavity. This provides a means for increased suction as prey, such as zooplankton, are consumed out of the open water. The large adaptive radiations of African cichlids present such patterns of divergence in the preorbital region whereas “ecomorphs” belonging to cases of adaptive divergence are also found to display such changes within many species of postglacial fishes such as pumpkinseed and bluegill sunfish (*Lepomis gibbosus, Lepomis machrochirus*), salmonids, European perch (*Perca fluviatilis*), and three‐spined sticklebacks (*Gasterosteus aculeatus*; Skúlason et al., 2019; Figure 1).

These broadly consistent patterns of adaptive divergence in the preorbital region of fishes have influenced a number of studies exploring the plastic responses to benthic and limnetic foraging conditions (Robinson & Parsons, 2002; Skúlason et al., 2019). Indeed, across several species and populations, plastic responses are broadly similar to benthic treatments inducing a reduced jaw length and a deeper, shorter body relative to limnetic treatments (Figure 1). These induced responses have also been demonstrated as adaptive in that they can significantly improve foraging efficiency (Andersson, 2003; Day & McPhail, 1996; Parsons & Robinson, 2007). Notably, evidence suggests that these patterns of plasticity, particularly in the craniofacial region, are aligned with broader patterns of adaptive divergence in both cichlids and sticklebacks (Wund, Baker, Clancy, Golub, & Foster, 2008; Parsons et al., 2016; Wund, Valena, Wood, & Baker, 2012). The stickleback example is especially powerful as it demonstrates that populations of extant putative ancestors (i.e., marine sticklebacks) exhibit plastic responses with strong similarity to current benthic and limnetic ecotypes occurring repeatedly across independent lakes. This suggests that plasticity can immediately initiate the direction of longer‐term subsequent evolution in response to new environmental conditions. These observations in sticklebacks are consistent with what is referred to as the “flexible stem” hypothesis (see West‐Eberhard, 2003), which is gaining interest from researchers especially in light of ideas such as “plasticity‐first” evolution (Levis & Pfennig, 2016).

The flexible‐stem hypothesis posits that if an ancestral stem group repeatedly colonizes similar environments, developmental plasticity should give rise to similar phenotypes. Subsequent adaptation can then arise from the selection on these phenotypes meaning that plasticity sets the initial course for evolution. Such a process could provide an explanation for the often‐repeated nature of adaptive divergence (i.e., parallel evolution). This also suggests that plasticity can initiate phenotypic biases through the similar initial responses of ancestral populations. However, our best evidence of the flexible stem, which comes from the stickleback example (Wund et al., 2008), may be more complex than originally thought. While marine sticklebacks have repeatedly colonized and evolved in freshwater habitats, there has been genetic exchange over time. Indeed, whole‐genome evidence shows that freshwater sticklebacks are more similar to their nearest ocean‐dwelling counterparts, and so‐called “freshwater alleles” persist in marine populations at low frequency (Jones et al., 2012). Further, these alleles are subject to gene flow within marine populations providing an explanation for how the genetic changes involved with adaptation to freshwater are similar at a global scale (Jones et al., 2012). Therefore, the genetic variation present in the putative marine ancestors of freshwater sticklebacks in Wund et al. (2008) could have contained alleles and developmental elements previously favored in past freshwater environments. This raises the question of whether the flexible stem represents a form of developmental bias that arises within “evolutionarily familiar” environments (sensu Bondurianskay & Day, 2018). Below, we discuss how developmental systems may enable such biases to arise through phenotypic plasticity.

## 3 DEVELOPMENTAL SYSTEMS CAN PROVIDE BIASED PLASTICITY

Although plasticity is often defined in relation to the genotype, a developmental perspective would suggest that it represents the interpretation of a developmental system to environmental cues. Undoubtedly, the interpretation of environmental cues by development is influenced by genetic variation, which in turn can be determined by selection but also evolutionary history. By evolutionary history, we mean past events that have affected trait variation and its potential for emergence within a lineage. While such evolutionary history may not initiate divergence, it can facilitate and act as a guiding force for what phenotypes are obtained from ongoing evolutionary drivers. A tactic that was perhaps used by some to help phenotypic plasticity gain acceptance in evolutionary biology was to describe it as a “trait” just like any other, in that it possesses heritable variation and can be influenced by selection (Schlichting & Pigliucci, 1998). In line with this, we suggest that plasticity is like other phenotypic traits in that it can also reflect evolutionary history (i.e., phylogenetic effects). Indeed, many well‐known vestigial traits persist because of past adaptive value and the inability of selection to remove them due to a lack of genetic variation or strong trade‐offs in developmental systems (Smith et al., 1985; West‐Eberhard, 2003). Alternatively, vestigial traits may persist because they are effectively neutral in relation to fitness and thus no longer the target of selection. However, vestigial traits do not necessarily need to exist as outward physical structures (Kijimoto, Moczek, & Andrews, 2012; Moczek, 2008). Instead, developmental systems can harbor past developmental programs that effectively become phenotypically “silent” through evolutionary processes or changes in environmental conditions (i.e., cryptic genetic variation, hereafter CGV; Gibson & Dworkin, 2004). It is important to note that CGV is normally thought to accumulate through mutations that arise but are phenotypically silent, due to developmental processes such as canalization. Such mutation‐driven CGV is more likely to produce random variation than the alternative way, whereby formerly adaptive developmental programs are silenced by development.

**Figure 2:**
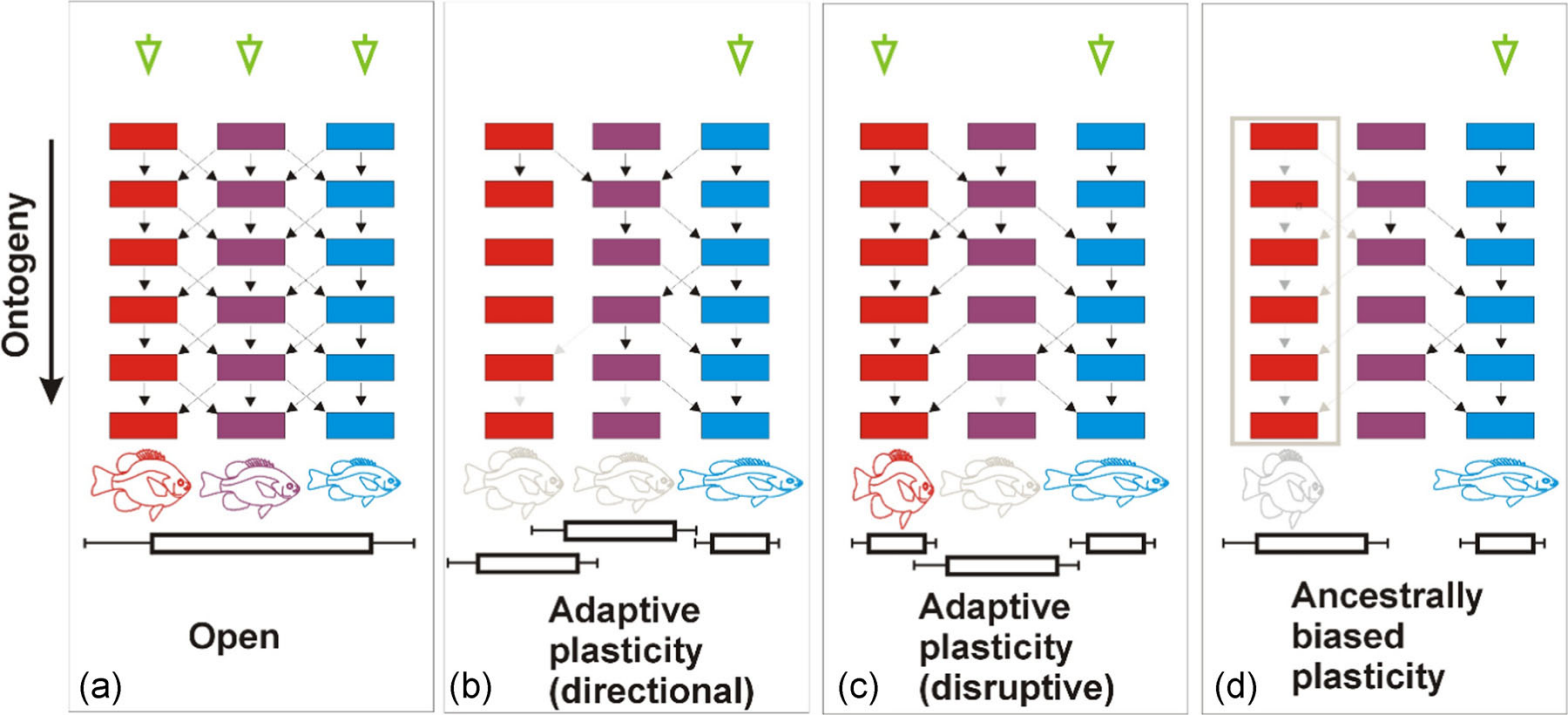
Four scenarios (a–d) for how systems with three developmental pathways (red, purple, and blue) may respond to environmental inputs (green arrowheads). In (a), an open developmental system exists whereby any possible phenotype can be produced by any type of environmental input. There are numerous connections between elements of the pathways (colored blocks), allowing for a high degree of flexibility. The outcomes of these processes are likely to be highly variable (indicated by error bars) and thus provide a high degree of evolutionary potential. Though these phenotypes can provide adaptive variation, they are unrefined responses that can be improved through selective processes that alter developmental systems. In (b), a developmental system is presented that has been exposed to a single and constant environmental input. This environment has selectively favored a directional adaptive response, which is reflected in a loss of connections between pathway elements that are infrequently used or cause antagonistic pleiotropy. This has resulted in a more refined phenotypic response (note the more extreme body shape relative to the blue phenotype in (a)). Further, while the other phenotypes are still possible, the adaptive process has resulted in a relatively small degree of phenotypic variation for the most frequently expressed phenotype. The differences between (a) and (b) may be representative of processes such as genetic accommodation or genetic assimilation. Similarly, in (c), connections between pathway elements have been lost to optimize the frequency of two phenotypes in response to two distinct environmental inputs. These inputs could occur simultaneously or as an oscillating system that both favor the refinement of two phenotypes (reflected in more‐extreme body shapes of the red and blue phenotypes) with low levels of variation. Finally, in (d), a shift in environmental parameters has favored the development of a single phenotype but the connections for the ancestral adaptive phenotype remain latent in the developmental system (gray arrows). In this case, developmental trade‐offs and antagonistic pleiotropy are environmentally dependent and avoided over evolutionary time by stable environmental cues that favor a single phenotype different from the “potential” phenotype (gray). The latent program can be reactivated by a further shift in environmental inputs (or mutation) and elicit a phenotype that reflects the past adaptation. However, the re‐emerged phenotype possesses a high degree of variation due to the loss of refinement over evolutionary time whereas these aspects of the developmental system were silent and effectively neutral to selection [Color figure can be viewed at wileyonlinelibrary.com]

When such formerly adaptive developmental programs are silenced they are no longer the direct target of selection. These programs can then be triggered to reproduce an ancestral phenotype by a change in environmental conditions or even a mutation. An example of this can be found within ants belonging to the *Pheidole* genus (Rajakumar et al., 2012). In these ants, the caste system typically comprises a queen, soldier, and minor worker. However, in some species, an additional caste referred to as a “supersoldier” exists. This caste serves a unique role in the colony in that it possesses a larger head relative to other castes, which it uses to block the entrance to the nests during army‐ant raids. This supersoldier caste is relatively rare within this genus but was known to be present in two phylogenetically distant species separated by over 35 million years of evolution. Recent comparisons of embryos from different castes revealed that supersoldiers showed a relatively high level of expression in the *sal* gene particularly in areas of wing disc development. While the supersoldier does not possess functioning wings, it does obtain small vestigial structure‐resembling wings. Impressively, experiments using methoprene (a mimic of the juvenile hormone naturally differentially emitted by the queen to induce different castes) at specific points in development could recapitulate the supersoldier caste in a third of tested species where it did not naturally exist (Rajakumar et al., 2012). This means that the developmental system retained the potential for this caste that was reignited by plasticity. Such a plastic response, bringing about a complex phenotype after millions of years of absence, strongly suggests that plastic responses and cryptic genetic variation can be nonrandom. Also, it could be that adaptive plasticity in early generations of a lineage invading a new habitat is actually reflective of a “rerelease” of a phenotype that has previously faced selection (Figure 2). The evolutionary timeframe in *Pheidoles* suggests that such an effect can last for millions of years.

Therefore, in some cases, the flexible stem could be viewed as a biased ancestral plastic response that reflects evolutionary history in a lineage. For example, in Wund et al. (2008), it could be that populations of marine sticklebacks have responded to diet treatments similarly because they represent “tried and tested” phenotypes produced by development in evolutionarily familiar environments (Bondurianskay & Day, 2018). Notably, Wund et al. (2008) did not observe a loss of plasticity in their derived freshwater populations. This means that any gene flow with oceanic populations would include the genetic basis for such adaptive plasticity. Such directed responses could explain the seemingly rapid adaptation of some populations of sticklebacks and other species. Further, it could be inferred that adaptative evolution is ultimately a slower process than currently thought, including cases of contemporary evolution (see Stockwell, Hendry, & Kinnison, 2003), in that accumulated adaptations from the past are not necessarily erased by current selection regimes. Thus, many adaptations that arise rapidly may have an advanced start due to the ability of developmental systems to hide them intermittently and use them when they will confer advantages. This implies that input signals need to be properly interpreted for a developmental system to provide the appropriate output in the form of an adaptive response (Figure 2).

Obtaining a developmental system that provides an appropriate plastic response (i.e., adaptive plasticity) should involve standard processes of selection on realized phenotypes. Further, the frequency with which different phenotypes are realized by plasticity and face selection should also determine the rate for adaptive plasticity to evolve. However, when we consider the underlying mechanisms of development, it could be that some elements that determine adaptive responses in one environment also provide maladaptive responses in another. For example, in a mechanistic context, this could arise due to interactions within members of molecular signaling pathways that are utilized for adaptive phenotypes in multiple environments. If the selection in one inductive environment favors changes that downregulate the pathway under current conditions, and if this change also downregulates the pathway in another frequently encountered environment where upregulation is favored, it would confer trade‐offs in the developmental system that appear across plastic responses. Therefore, the selection on plasticity in one environment may create maladaptive plasticity in others. This may not be an issue when organisms live in stable environments over generations as it would allow adaptive plasticity to evolve in a single direction (with alternate plastic phenotypes only rarely being realized). However, we know that in many organisms, environmental conditions fluctuate frequently and would likely generate selection specific to a given condition (Ledón‐Rettig et al., 2014). This is evidenced by some populations where plastic responses result in a bimodal phenotypic distribution that matches current conditions adaptively (Levis & Pfennig, 2016). In such cases, cross‐environment developmental trade‐offs in plasticity may be minimized by selective processes. But how could such trade‐offs be avoided at a mechanistic level?

We suggest one way that trade‐offs mediated by plasticity can be avoided is through the evolution of alleles that are exclusive to a given environment. This would result in a genetic architecture for traits that are determined by environmental conditions rather than phenotypes being predetermined by genetic variation. In line with this idea, our recent findings using QTL mapping in an African cichlid pedigree show that the genetic basis of morphological traits is largely determined by foraging environment (Parsons et al., 2016). Indeed, morphological differences were induced by feeding cichlid groups either a benthic or limnetic prey mimic consisting of the same nutritional value. These differences included the usual lengthening of the oral jaws (i.e., preorbital region) under limnetic treatments, relative to the shortening of the jaws in the benthic treatments. Of the 22 QTL identified for various traits, only one was present across environments relating to the mechanical advantage of the jaw. However, the alleles related to this single cross‐environment QTL showed differential sensitivity to benthic and limnetic foraging suggesting that trade‐offs could be avoided by reducing the phenotypic effects of alternate allelic states. Therefore, it seems that plasticity can be especially effective at silencing the effects of even adaptive alleles in a way that avoids pleiotropy or “crosstalk” between phenotypes that are induced in alternate environments. If this is a general occurrence, this finding provides a number of important implications for how developmental bias may be impacted. First, it suggests that bias resulting from the evolution of adaptive plasticity in past environments can be largely mitigated by a wholesale change in the genetic architecture of trait variation in the current environment. Second, this finding also suggests that plastic responses are not primarily based on “plasticity genes” whereby distinct loci operate across environments by possessing environmentally sensitive alleles (sensu Pigliucci, 2001; Scheiner, 1993). Third, the determination of the genetic architecture by the environment may negate the impact of additive genetic variation (at least in early generations). In other words, the inheritance of an allele from a parent, even adaptive ones under their given environment, may be of little or no consequence under the environmental conditions of the offspring. Combined, these aspects could make plasticity especially effective at providing novel genetically based variation that avoids developmental trade‐offs and, in turn, avoids the effects of a genetically programmed developmental bias.

However, at the same time, this ability of plasticity to readily hide the genetic mechanisms of adaptive phenotypes could also protect them from loss due to selection. This could provide a means for biased responses to arise in current environments, relating to both concepts of cryptic genetic variation which is commonly thought to accumulate through neutral processes under “normal” environmental conditions (Paaby & Rockman, 2014), and the flexible‐stem hypothesis (West‐Eberhard, 2003). Conversely, the release of cryptic genetic variation is thought to occur under atypical environmental conditions and facilitate adaptation through conventional selective processes if “by chance” adaptive variation is generated (Ghalambor, McKay, Carroll, & Reznick, 2007). Thus, the idea of CGV largely holds to the same convention of general mutations in that variation is created randomly to facilitate the creation of phenotypic variation. Instead, we suggest that CGV can also accumulate as a result of past evolution and serve as a reservoir of adaptive outcomes that reside in a latent state within the developmental system. Therefore, CGV and the induction of a flexible stem could represent the same developmental phenomena to some degree.

Instead of the emergence of random variation with environmental shifts (i.e., CGV), we suggest that the re‐emergence of an adaptive trait from a lineage's past is possible. Although such a trait may re‐emerge to be adaptive, it may not an be an optimum fit to current conditions (Figure 2.). This means that selection could still adjust plasticity to improve its adaptive value after an ancestral phenotype has re‐emerged. The degree to which such variation is expressed may then be determined by the time a lineage has spent in a given habitat, and how specialized it has become. Indeed, previous research on arctic charr (*Salvelinus alpinus*) ecomorphs shows that while they all display the typical shape responses to benthic and limnetic diets, the variance in shape corresponds to their degree of ecological specialization (Parsons, Skulason, & Ferguson, 2010). Specifically, more specialized benthic ecomorphs from multiple populations show substantially greater increases in shape variance when reared on a “nonnative” limnetic diet (Parsons et al., 2011, 2010). This is likely reflective of a developmental system that has not been exposed to such conditions for several thousand years. Thus, it seems the mechanisms determining biased plastic responses that have also been selected on in the past can persist, whereas other changes can indeed accumulate through neutral processes until presented to selection by a change in developmental conditions. Therefore, we suggest that not all variation interpreted as CGV is the result of neutral processes, rather there is a mixture of past adaptive plasticity and accumulated variation that are both effectively neutral under certain conditions. This suggests that our current null expectation that CGV provides random phenotypic outcomes deserves further attention.

## 4 BIASED PLASTICITY, ADAPTIVE DIVERGENCE, AND ADAPTIVE RADIATION

Schluter (1996) provided the core theory of “genetic lines of least resistance” to address how the direction of evolution is determined. Within this quantitative genetic framework, the multivariate direction of greatest additive genetic variance (*g*_max_) represents the path of least resistance for adaptation. By determining *g*_max_ for a population, it was widely thought that one can predict evolutionary outcomes over long time scales. However, evidence indicates that such predictions from quantitative genetic approaches often hold for only a single generation (Pigliucci, 2006). Nonetheless, the concept of genetic lines of least resistance remains appealing to many researchers as cases of adaptive divergence often exhibit stereotypical patterns whereby parallelism or convergence in phenotypes predominate across populations or species (Dick, Hinh, Hayashi, & Reznick, 2018; McGlothlin et al., 2018). This was thought to occur because lineages with shared ancestry in turn share *g*_max_ (or more generally standing genetic variation) and therefore evolve similarly (Thompson, Osmond, & Schluter, 2019).

However, adaptive divergence and adaptive radiations are also predicted to occur when shifts in environmental parameters occur (Schluter, 2000). Inherently, such shifts would inevitably induce new phenotypic variation through plasticity, and based on our evidence, a wholesale shift in the genetic basis of adaptive phenotypic variation (Küttner et al., 2014; Parsons et al., 2016). In this way, adaptation could be considered to take place along “developmental lines of least resistance” whereby the path of evolution is determined by plasticity. This means the effect of additive genetic variation would be minimized upon exposure to a new environment and therefore not play a role in initiating divergence. The *g*_max_ of subsequent generations would be realized by stable environmental cues. Therefore, biases in plasticity (such as similar responses to a range of environments) arising from the release of CGV could be especially important for generating adaptive divergence and radiations.

In some cases, environmental shifts may represent an oscillation between a limited number of environmental conditions over time. Indeed, the *Anolis* lineage comprises more than 300 species that have only evolved four primary ecotypes over 30–40 million years (Langerhans, Knouft, & Losos, 2006; Losos, 2007). Similarly, damselfishes (Pomacentridae) also comprising nearly 300 species have diverged into only three primary ecotypes. Convergence in the morphological and kinetic function of the jaws has been especially important for damselfish with rapid and repeated ecological shifts characterizing the evolution of this lineage over 50 million years (Cooper & Westneat, 2009; Frédérich, Sorenson, Santini, Slater, & Alfaro, 2013). Could this lack of expansion into truly novel niches be due to biases in phenotypic plasticity? Regarding *Anolis*, we know so far that plasticity occurs in hindlimb length in relation to perch diameter, whereby legs lengthen in response to a broad substrate but shorten in response to narrow surfaces (Kolbe & Losos, 2005; Losos et al., 2000). These plastic responses are likely adaptive based on well‐established relationships in *Anolis* including among populations of some species and among species (Irschick & Losos, 1998; Losos & Irschick, 1996). Notably, both *Anolis sagrei* and *Anolis carolinensis*, which are distant relatives, respond similarly to perch size treatments suggesting that hindlimb plasticity is widespread in the genus and evolved within a common ancestor (Kolbe & Losos, 2005). For damselfish, we are unaware of research suggesting plasticity in trophic morphology but would expect that they follow patterns of responses found across a wide range of fish species (Parsons et al., 2016; Robinson & Parsons, 2002; Skúlason et al., 2019).

**Figure 3:**
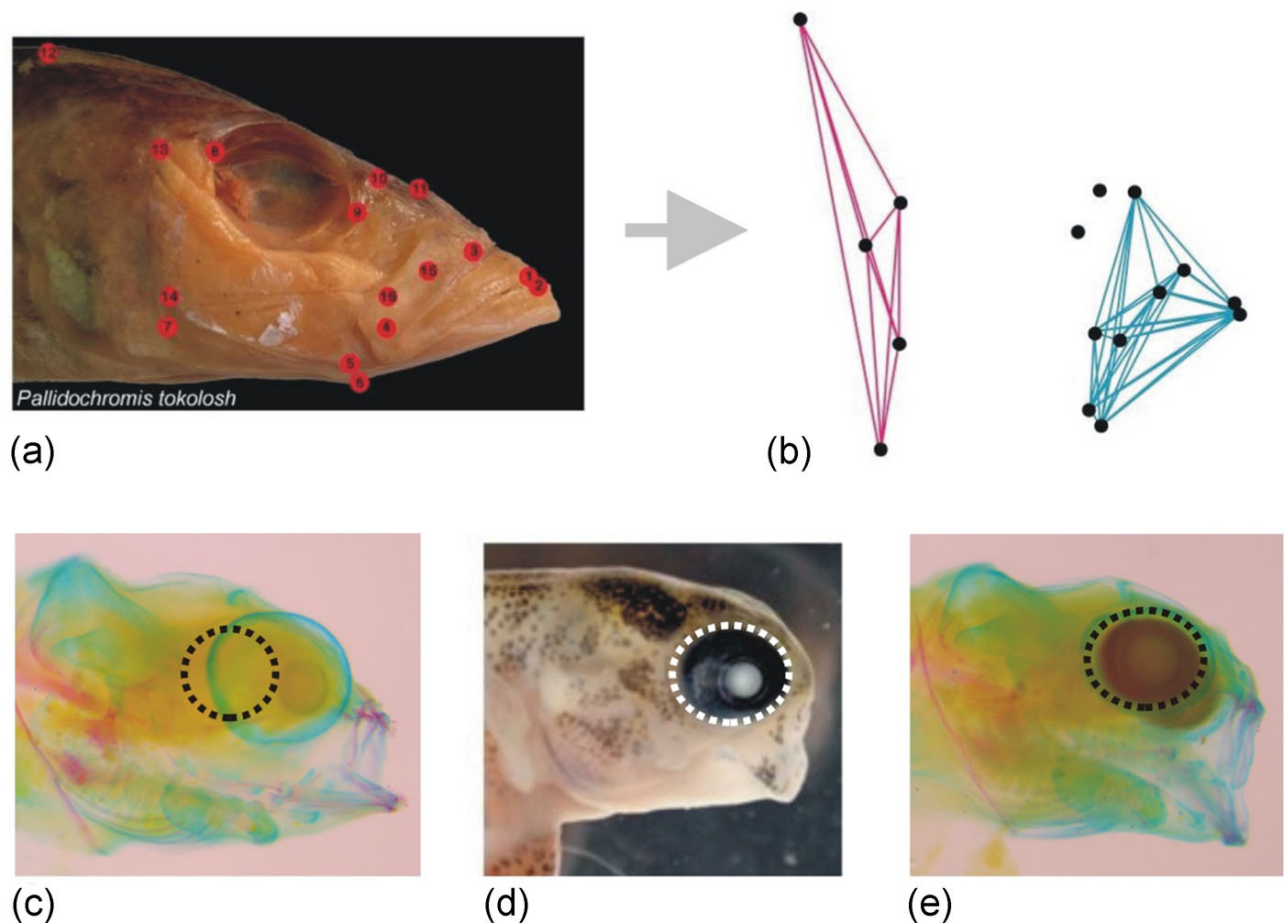
Potential mechanisms of developmental bias in the preorbital region of Malawi cichlids could lie in patterns of modularity (a, b), and the reliance of phenotypic variation on a limited number of developmental pathways (c, d). (a) Depiction of the landmarks used to assess both covariance and shape in the craniofacial region of over 80% of the genera of African cichlids from Lake Malawi (from Cooper et al., 2010). The preorbital region (blue lines connecting landmarks) comprises a distinct variational module in Malawi cichlids (b). The projection of plastic variation on such a “scaffold” for development lends itself well to the observations of bias in the preorbital region (see Figure 1). Further, the projection of variation on a developmental system that relies on few pathways would be more likely to result in similar phenotypes. This is seen in the similar production of a “lockjaw” phenotype in *Labeotropheus fuelleborni* embryos (control group = C) in response to lithium chloride (d), which is an agonist of Wnt/β‐catenin signaling (from Parsons, Trent Taylor, Powder, & Albertson, 2014), as well as to bepridil (e), which is an inhibitor of calcium signaling (Walker, McWhinnie, and Parsons, unpublished). These relatively similar phenotypic responses to different molecular targets suggest that craniofacial development in cichlids is developmentally limited with particularly strong effects on the preorbital region. Note that the orbital bone has been highlighted in these pictures, and that swelling of the eye has occurred due to staining and preservation methods [Color figure can be viewed at wileyonlinelibrary.com]

The repeatability of adaptive radiations could be influenced by how plasticity interacts with trait covariance. Trait covariance can arise for a number of reasons, such as shared developmental origins during early stages of ontogeny (e.g., traits derive from a common cell population), or the functional coupling of traits. Such trait covariance is prevalent in adaptive radiations (Felicea & Goswami, 2017; Parsons, Márquez, & Albertson, 2012) and may serve as a “scaffold” upon which phenotypic variation is projected (Parsons et al., 2018). In support of this, our findings of craniofacial plasticity in Malawi cichlids show that the oral jaws undergo greater change than other regions in response to benthic and limnetic foraging (Figure 1; Parsons et al., 2016). This coincides with independent evidence that the oral jaws comprise a separate variational module that is distinct from the remainder of the head across all three of the major cichlid‐adaptive radiations in lakes Malawi, Victoria, and Tanganyika (Figure 3; Parsons et al., 2012). Thus, while plasticity occurs in response to foraging treatment, the variation generated could be funneled through such patterns of trait covariance to create more localized and biased responses. However, this interpretation presumes that patterns of covariance and variational modularity are static during plastic responses. Although still not well understood, it has been documented several times that patterns of trait integration can change in response to environmental conditions, possibly as a result of differential sensitivity of traits (Ellers & Liefting, 2015; Handelsman, Ruell, Torres‐Dowdall, & Ghalambor, 2014; Peiman & Robinson, 2017). Nevertheless, it may still be that static processes play an important role in directing plastic variation because integration ultimately reflects the layering of multiple proximate determinants of covariance over ontogeny (see Hallgrímsson et al., 2009).

Plastic responses themselves can covary and likely lead to biased plastic and, in turn, evolutionary responses. Referred to as plasticity integration, the reaction norms of several individual traits can respond together in a coordinated way that may improve fitness (Schlichting & Pigliucci, 1998). Such integration may have long‐term evolutionary effects as it appears to be conserved in both life history and wing morphological traits across species of mycalesine butterflies in response to temperature. This conservation of responses in butterflies occurs despite considerable periods of independent evolution in widely different environments (van Bergen et al., 2017). However, plasticity integration can vary both among populations and genotypes, suggesting it can evolve rapidly (Plaistow & Collin, 2014). Indeed, the adaptive divergence between benthic and limnetic ecomorphs of pumpkinseed sunfish is associated with changes in plasticity integration. In this case, patterns of plasticity integration converge in multiple instances of divergence in independent lakes with general increases in the number of integrated reaction norms occurring in the more derived pelagic ecomorphs (Parsons and Robinson, 2006). This suggests that selection may favor biased patterns of plasticity through plasticity integration that generates a concerted and coordinated phenotype response across a range of traits. Possibly, this bias may negate some of the potential (and empirically elusive) costs of maintaining a system that senses and interprets environmental cues (van Buskirk & Steiner, 2009). This is because, fewer or perhaps only one trait requires an environmental signal to be interpreted by development to initiate an integrated set of adaptive plastic trait responses. At present, plasticity integration is a rarely explored topic, but these insights suggest that it may be especially important for initially forming and maintaining biases in plasticity and evolution. This is especially relevant in light of the idea that plasticity initiates adaptive divergence (Levis & Pfennig, 2016; West Eberhard, 2003).

## 5 RECOGNIZING BIASED PLASTICITY PHENOTYPICALLY AND WITHIN DEVELOPMENTAL SYSTEMS

Wider recognition and the identification of biased plasticity could lead to a deeper understanding of its mechanistic basis and impact on evolution. We suggest that bias in plasticity could be inferred from patterns in two main ways: (a) As similar responses to the same environmental cues across lineages and populations, or (b) as similar responses to dissimilar environmental cues if biases are more extreme. It is commonly appreciated that a wide range of species will often show similar responses to the same environmental cues (Robinson & Parsons, 2002; Martinez‐Garcia et al., 2014). For example, in centrarchid fishes, resource polymorphisms are known to exist in both pumpkinseed and bluegill sunfish whereby individuals within a single lake adaptively diverge into specialists that focus on either benthic or limnetic habitats with corresponding changes in head and body shape (Ehlinger & Wilson, 1988; Robinson, Wilson, Margosian, & Lotito, 1993). For pumpkinseed, these polymorphisms are known to occur repeatedly across several populations in Ontario, Canada, as well as in the Adirondacks of New York State, which respectively form two lineages (Weese, Ferguson, & Robinson, 2012). Experiments show that pumpkinseeds from different lakes and ancestral lineages respond in a similar way to benthic and limnetic diet treatments (Parsons & Robinson, 2006, 2007; Robinson & Wilson, 1996; Weese et al., 2012). Further, similar changes in response to the same type of diet treatments are also observed in the orange‐spotted sunfish (*Lepomis humilis*; Hegrenes, 2001). Taken together, we can then infer that some aspects of plastic responses are likely biased at the genus level in sunfish and that these persist across historical lineages and populations. Also, given that two species of sunfish (bluegill and pumpkinseed) are known to exhibit similar resource polymorphisms that mirror the direction of plastic benthic/limnetic responses we can suggest that ancestrally biased plasticity is influencing their evolution (i.e., the flexible stem). An intriguing question is whether these responses are based on the same mechanisms across species and populations. Such information could be used to examine the presence, persistence, and function of alleles and developmental interactions across taxonomic levels. Indeed, much of the similarity in these responses could simply be due to similar patterns of biomechanical stress and a common developmental response that has evolved ancestrally (Conith, Lam, & Albertson, 2019).

Genetic variation for any trait does not act in isolation of a developmental system. Therefore, instead of bias in plasticity being caused by shared allelic variation, it could instead be due to the reliance of phenotypic variation on a limited set of developmental pathways. This idea has justification from several evo–devo studies showing that similar phenotypes are underlain by different genes that belong to a shared set of signaling pathways (Kingsley, Manceau, Wiley, & Hoekstra, 2009; Nachman, Hoekstra, & D'Agostino, 2003; Steiner, Weber, & Hoekstra, 2007). For example, morphological variation in the craniofacial apparatus of vertebrates is largely found to include genes belonging to five primary signaling pathways (Parsons & Albertson, 2009; Parsons, Wang, Anderson, & Albertson, 2015). These five include the Wnt/β‐catenin, fibroblast growth factor (FGF), transforming growth factor β, hedgehog, and Notch signaling pathways. While these signaling pathways cannot account for all morphological variation, evidence suggests that they are major players. Specifically, some of these pathways can be involved with the lengthening and shortening of the oral jaws in fishes and other vertebrates (Parsons & Albertson, 2009; Parsons & Albertson, 2013). Our own research on the genetic basis of plasticity has shown that plasticity in the mechanical advantage of the jaw in cichlids is in part due to hedgehog signaling (Parsons et al., 2016). If other organisms follow a similar result with plasticity (like other traits) being largely reliant on a few sets of signaling pathways, there are important implications for its potential bias. Notably, relying on a few developmental pathways could substantially reduce the degrees of freedom for phenotypic development and adaptation. Such primary pathways, if sensitive to the environment, would, in turn, induce a limited number of phenotypic outcomes from plasticity. Given how genes interact intimately within such pathways (often with several interdependencies), the phenotypes based on them are likely a broader target for adaptive evolutionary change (see Steiner, Rompler, Boettger, Schoneberg, & Hoekstra, 2009). In other words, there may be more than one way for selection to alter a pathway through allelic change at multiple loci, but perhaps with only a limited number of phenotypes (creating bias). Future research examining the mechanisms of plasticity should reveal which pathways are involved most frequently. It may be that some pathways are particularly sensitive to environmental cues (e.g., hedgehog signaling from Parsons et al., 2016) and it would be especially interesting to determine whether these pathways are also the same as those commonly implicated in adaptive divergence (Parsons & Albertson, 2009, 2013). Such a finding would also support the idea that plasticity promotes evolution (but may also bias it).

Similar plastic responses to dissimilar environmental cues would be especially strong evidence of bias in plasticity as they would indicate the existence of a predetermined developmental program exists. This again could be due to the reliance of phenotypes on a limited number of signaling pathways. Our preliminary and other existing evidence suggests that such a scenario is possible with regard to jaw length in fish. Specifically, sticklebacks respond to benthic and limnetic diet treatments as would be expected with a respective shortening and lengthening of the preorbital region (Figure 1; Wund et al., 2008). However, sticklebacks also respond to temperature treatments in a similar manner with longer jaws being induced by increases in temperature and shorter jaws occurring in lower temperatures (Figure 1; Pilakouta et al., 2019; Ramler, Mitteroecker, Shama, Wegner, & Ahnelt, 2014). How could such a result emerge? There are a number of possibilities, with the simplest being that adaptive plasticity has evolved similarly for both environmental gradients. However, it could also be due to an evolutionary history whereby the developmental system has been repeatedly exposed to similar environmental cues. In the case of fishes, based on their propensity to lengthen and shorten their oral jaws during adaptive divergence, it could be inferred that their evolutionary history reflects repeated exposure to benthic and limnetic habitats. This oscillation between these two types of habitats would be predicted to lead to the evolution of a developmental system that is biased and most frequently produces either a benthic or limnetic phenotype. Given the evolutionary success of fishes, this strategy is likely favored over evolutionary time as it improves fitness in most cases of habitat change (see Figure 2c). On the contrary, in response to truly novel or rarely encountered environmental conditions, such changes may still be induced even when they are maladaptive. This again would be akin to CGV but with the caveat that plastic responses are shaped by past selection, and the genetic variation involved with them has not always been cryptic. We suggest this idea of biased CGV could be addressed through new approaches to plasticity experiments. Specifically, for multivariate traits where trajectories can be readily quantified, plastic responses of different species or ecotypes could be compared against the main trajectories of adaptive divergence to reveal bias. Further, to demonstrate biased responses we also suggest that plastic responses should be induced by a range of habitat gradients to investigate similarities in the response. This approach may be particularly relevant for populations experiencing truly novel habitats relative to their evolutionary history (e.g., toxins, extreme temperatures).

## 6 SUGGESTIONS FOR FUTURE RESEARCH

Below, we have identified a number of areas that would be of interest to those investigating the role of plasticity in initiating and contributing to developmental bias. We provide three key questions to follow‐up on our ideas that could be used to motivate future research aimed at determining the presence and mechanisms of bias in plasticity. We also briefly outline some considerations, predictions, and caveats for each question that researchers should consider.

### 6.1 How unbiased or biased is a plastic developmental system?

We may predict that the degree of bias in plasticity will be reflected in the degree of variation exhibited in plastic responses (Figure 2). However, such variation should be measured within a single environment of a plasticity experiment. So far, plasticity research has mainly focused on a reaction norm perspective and measurements of the magnitude of plasticity across environments. Though important, this misses some further key insights into how a developmental system interprets environmental inputs. For example, the within‐environment phenotypic variation could be used to infer whether a relatively limited number of developmental interactions were used in a plastic response. In this case, reduced interindividual variation would suggest that fewer developmental interactions were used. Adaptive plasticity that has been refined by selection over generations should reflect a pattern of reduced variation, whereas plasticity, in response to a novel environment, should show wider phenotypic variation. Such thinking could be applied to both phenotypic and mechanistic studies.

### 6.2 How frequent is plasticity integration and how long does it persist?

Plasticity integration is rarely investigated but could be vital to reducing the potential costs of plasticity (but see Murren et al., 2015) and initiating developmental bias when organisms invade new habitats. In the context of adaptive divergence, we predict that plasticity integration is widespread and that its presence will be reflected in similar patterns of covariance among reaction norms across lineages. When plasticity integration is shown to evolve in an adaptive divergence, it could also be indicative of which traits first face selection and initially contribute to adaptation. More studies investigating plasticity integration are needed before we can fully interpret its role in evolution. Opportunities for this are quite plentiful given the number of researchers conducting plasticity experiments and that in many cases an additional analysis could reveal insights into plasticity integration.

### 6.3 What signaling pathways are involved with plasticity and how many?

A high degree of detail is needed to fully understand the dynamics of developmental pathways and can involve a career‐long endeavor (see Rothenberg, 2016). However, with modern sequencing techniques, it is now feasible to make rapid inroads by investigating transcriptome‐wide patterns of gene expression to reveal key pathways involved with plastic responses. Bias could be underlain by the reuse of a limited number of pathways across populations for the same trait and environment, the same pathways in different traits, or even the same pathways in responses to different environments. We suggest that researchers interested in biased plasticity could apply these and other approaches toward understanding pathway responses to environmental cues across populations and species (see Tobler, Kelley, Plath, & Riesch, 2018). This broader approach should be able to reveal patterns in the developmental pathways most commonly utilized in plastic responses. A straightforward prediction is that in cases of similar plastic responses across lineages, we should observe that they are caused by the expression of the same pathways in similar ratios. Further, while allelic changes underlying plastic responses may differ among lineages, they may result in functionally equivalent outcomes at the pathway and ultimately the phenotypic level. This perspective could have broad implications for those interested in phenomena such as parallel evolution. Specifically, in population genomic studies, an emphasis has often been placed upon the discovery of similar allelic changes (Jones et al., 2012; Miller, Roesti, & Schluter, 2019). Instead, many of the allelic changes that are unique to populations may contribute to parallelism (or similar plastic responses). This could be an especially important aspect of plasticity to understand, as it could initiate the evolution of such parallel changes (Wund et al., 2008).

## 7 CONCLUSIONS

How individuals and populations maximize their performance when facing environmental heterogeneity is of great interest to ecology and evolutionary biology. However, it is still taken for granted that variation for adaptive evolution is generated randomly. Although reflected in opinions of the role of mutation in evolution (which itself can be nonrandom), this view of a random process has perhaps subconsciously pervaded our views of phenotypic plasticity as a provider of variation. This could especially be the case given that plasticity is usually defined as a property of the genotype and is prevalent within our concepts of CGV and the selective process inferred for the evolution of adaptive plasticity. However, a more development‐based view recognizes the associated complexities involved with plasticity and why it may not generate variation in a random manner. For example, the developmental systems responsible for plasticity can reflect past evolutionary history and, in turn, limit the available responses to environmental inputs. Further, the evolution of adaptive plasticity may be associated with intermittent periods of selection and phenotypic expression. Adaptive plasticity also has the potential to be “stored” within developmental systems for considerable periods and re‐emerge in ways that produce biased phenotypic variation, which then guides the initiation of further evolution when environmental conditions change (e.g., the flexible stem). Thus, we should strongly consider the role of plasticity in both initiating and acting as a harbor for developmental bias.

## AUTHOR CONTRIBUTIONS

This manuscript was drafted by K. J. P. and critically edited by K. M. and N. P. Figures were provided by K. M., N. P., and L. W. and were derived from their ongoing research. K. J. P., K. M., and N. P. All read and approved the final draft of the manuscript.
